# Report of a case of cirrhotic portal hypertension with ectopic varices in the bilateral pulmonary hilar

**DOI:** 10.1002/deo2.99

**Published:** 2022-03-09

**Authors:** Ke Zhu, Wei Zhang, Wei Shao, Jing Ding, Chao Ma

**Affiliations:** ^1^ Department of Gastroenterology No.2 People's Hospital of Fuyang City Fuyang China

**Keywords:** ectopic varices, multidisciplinary team, portal hypertension, transjugular intrahepatic portosystemic shunt

## Abstract

Here, we report the case of patient cirrhosis with esophageal and gastric fundal varices who developed rare ectopic varices in the bilateral pulmonary hilar after repeated endoscopic treatments (tissue adhesive for gastric fundal varices + esophageal variceal ligation + esophageal variceal sclerotherapy) accompanied by serious shortness of breath. After transjugular intrahepatic portosystemic shunt + gastric coronary vein embolization, the shortness of breath was relieved, and the portography review indicated that the ectopic varices in the pulmonary hilar were significantly improved.

## INTRODUCTION

Ectopic varices are varices outside of the esophagus and the stomach.^1^ Currently, known ectopic varices mainly occur in the human digestive tract, for example, the duodenum, rectum, small intestine, and colon.^2^ With improvements in clinical diagnosis technology and ectopic varices awareness, ectopic varices have also been found in the retroperitoneum, abdominal wall, biliary tract, vagina, and bladder, albeit with a much lower incidence rate. With ordinary endoscopy, the detection rate of duodenal varices is 0.2% in general and 0.4% in patients with portal hypertension.^3^ Moreover, computed tomography (CT) angiography can help diagnose ectopic varices to some extent. In our case, the patient was ultimately diagnosed with ectopic varices in the pulmonary hilar through bronchoscopy and portography after multiple hospitalizations. However, varices in the pulmonary hilar have not yet been reported.

## CASE REPORT

**FIGURE 1:**
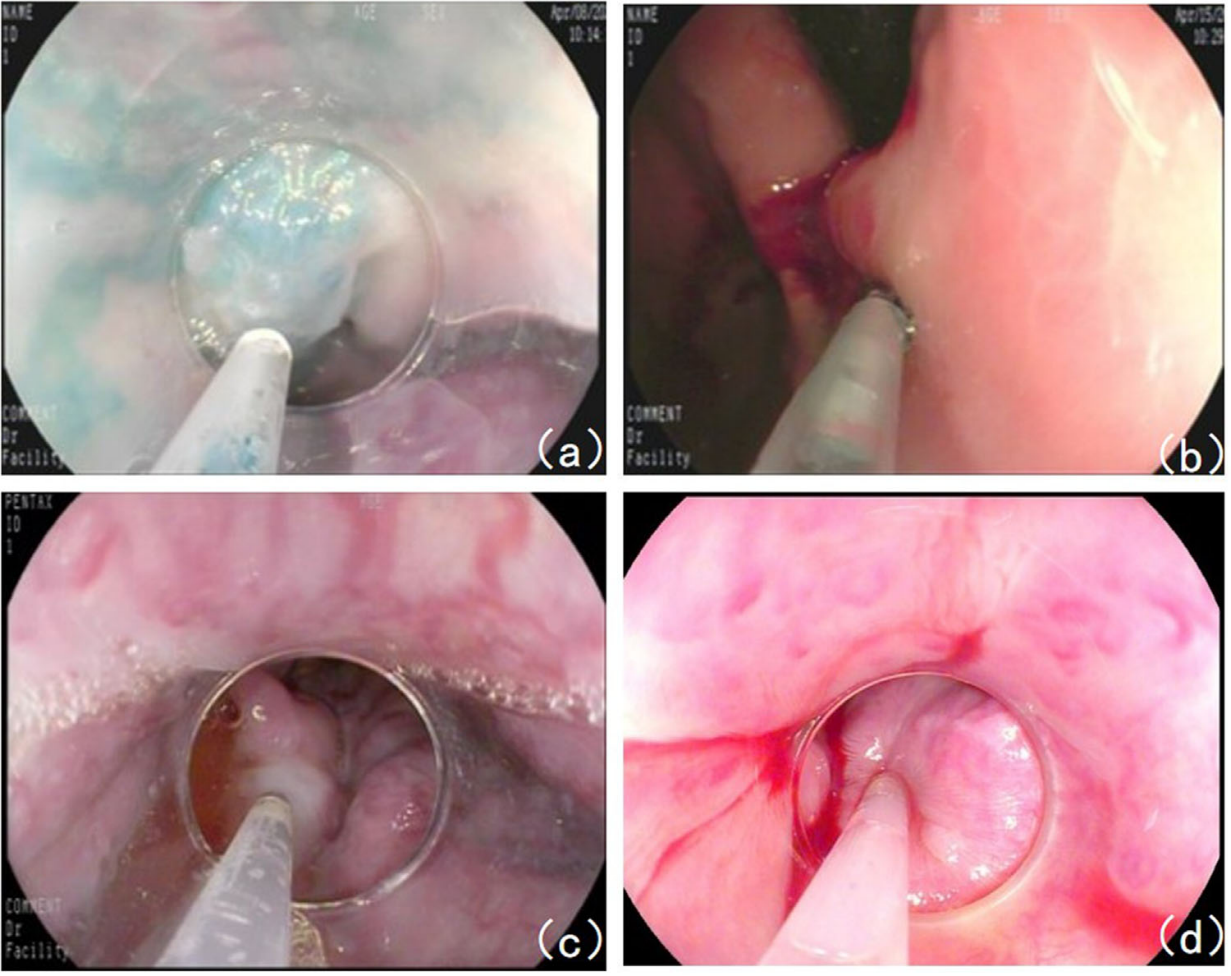
Endoscopic treatment of esophageal and gastric fundal varices: (a) Sclerotherapy for esophageal varices on April 8, 2020. (b) Sclerotherapy for esophageal and gastric fundal varices on April 15, 2020. (c) Sclerotherapy for esophageal varices on August 19, 2020. (d) Sequential sclerotherapy for esophageal varices on November 18, 2020.

**FIGURE 2:**
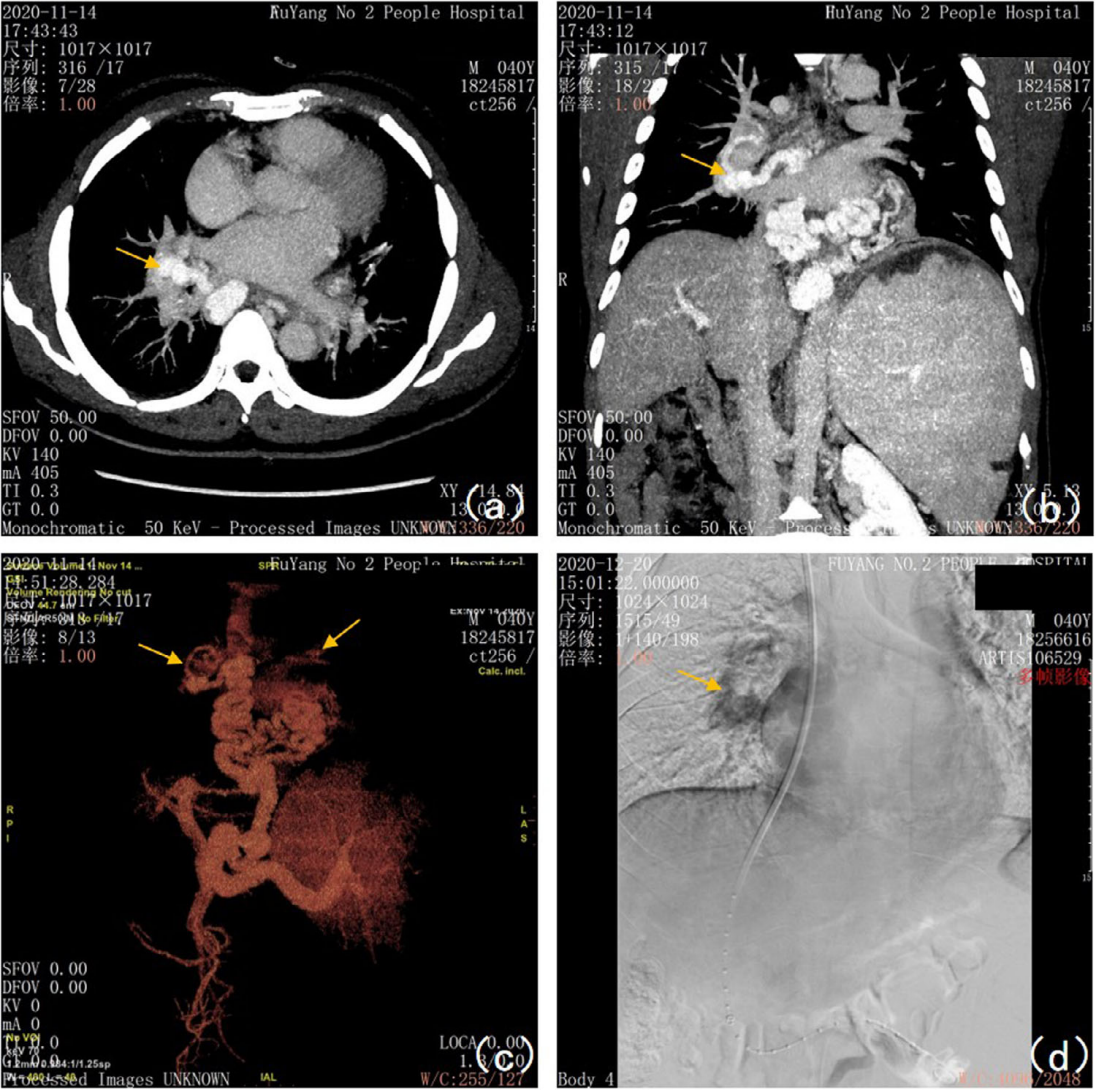
The manifestations of pulmonary hilar ectopic varices before transjugular intrahepatic portosystemic shunt under portal vein imaging on November 14, 2020, and X‐ray fluoroscopy: (a) The cross‐sectional scan of venous phase maximum intensity projection (MIP) image showed right pulmonary hilar ectopic varices (pointed by the yellow arrow in the figure). (b) The coronal scan of the venous phase MIP image showed right pulmonary hilar ectopic varices (pointed by the yellow arrow in the figure). (c) Portal venous reconstruction (VR) indicated thickened and tortuous esophageal and splenic veins, of which the wider lumen near the esophageal hiatus was 2.21 cm thick, and the main splenic vein was 1.99 cm thick, and tortuous vessels on both sides of the pulmonary hilar(pointed by the yellow arrow in figures) that extended downward to connect to tortuous vessels around the esophagus and partially converged with the main portal vein, with a lumen thickness of 2.73 cm near the confluence section. (d) Before the intrahepatic portosystemic shunt via the jugular vein, the pulmonary hilar varices (pointed by the yellow arrow in the figure) can be seen under X‐ray fluoroscopy with a contrast agent. The intraoperative hepatic venous pressure gradient was 39 cm H_2_O, and the direct portal vein pressure was 58 cm H_2_O before transjugular intrahepatic portosystemic shunt (TIPS)

**FIGURE 3:**
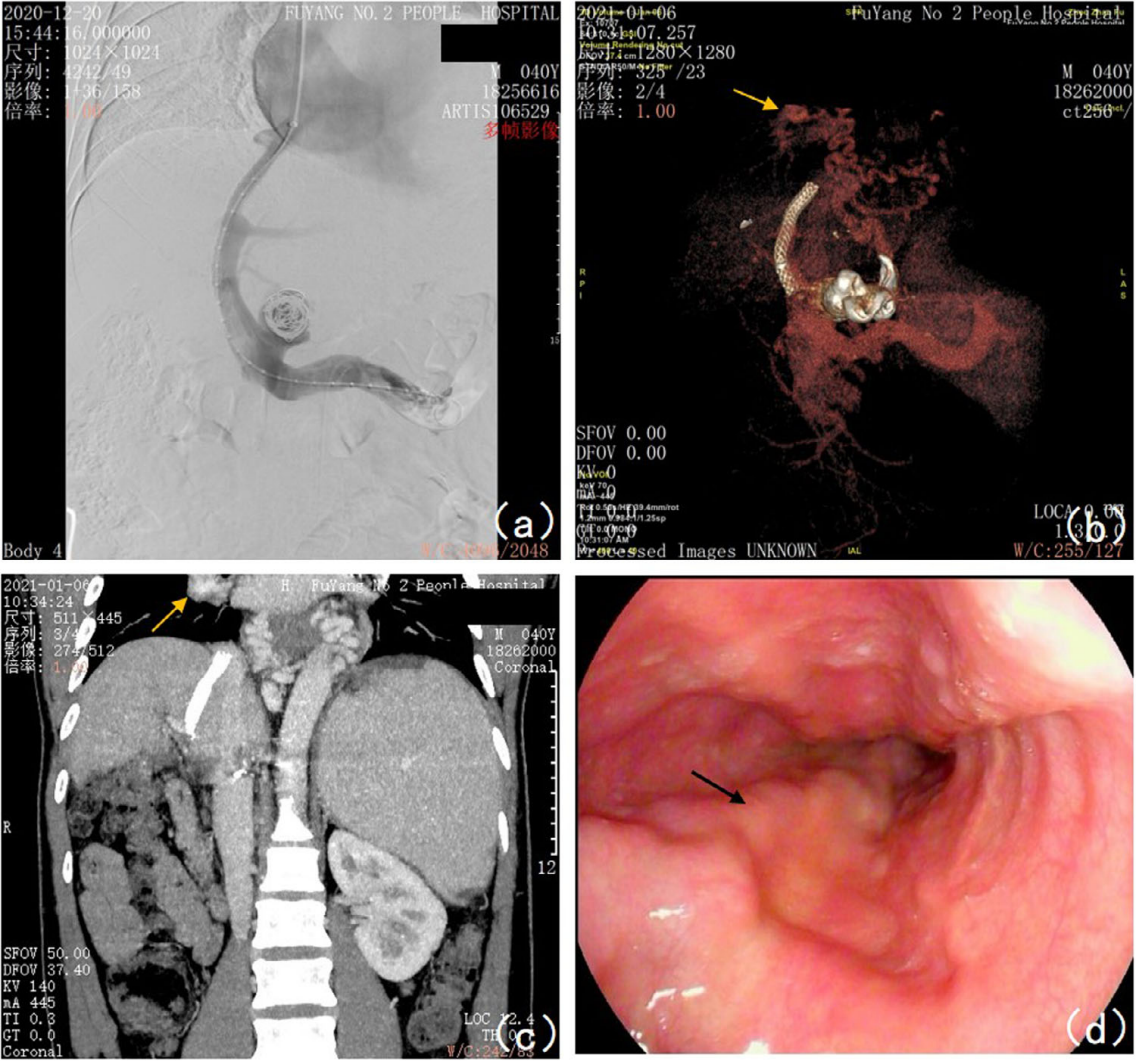
The manifestations of ectopic varices and esophageal varices after transjugular intrahepatic portosystemic shunt: (a) The direct portal vein pressure was 37 cm H_2_O after transjugular intrahepatic portosystemic shunt (TIPS). (b) Portography on January 6, 2021, showed post‐TIPS changes, a patent stent lumen, and the diameters of esophagogastric varices and pulmonary hilar ectopic varices were thinner diameters than before (pointed by the yellow arrow in the figure). (c) The coronal scan of the MIP image in the venous phase showed that the right hilar ectopic varices were thinner than before (pointed by the yellow arrow in the figure). (d) The diameter of esophageal varices was thinner than before, with red sign (‐) and blood bubble sign (‐) (pointed by the black arrow in the figure)

**FIGURE 4:**
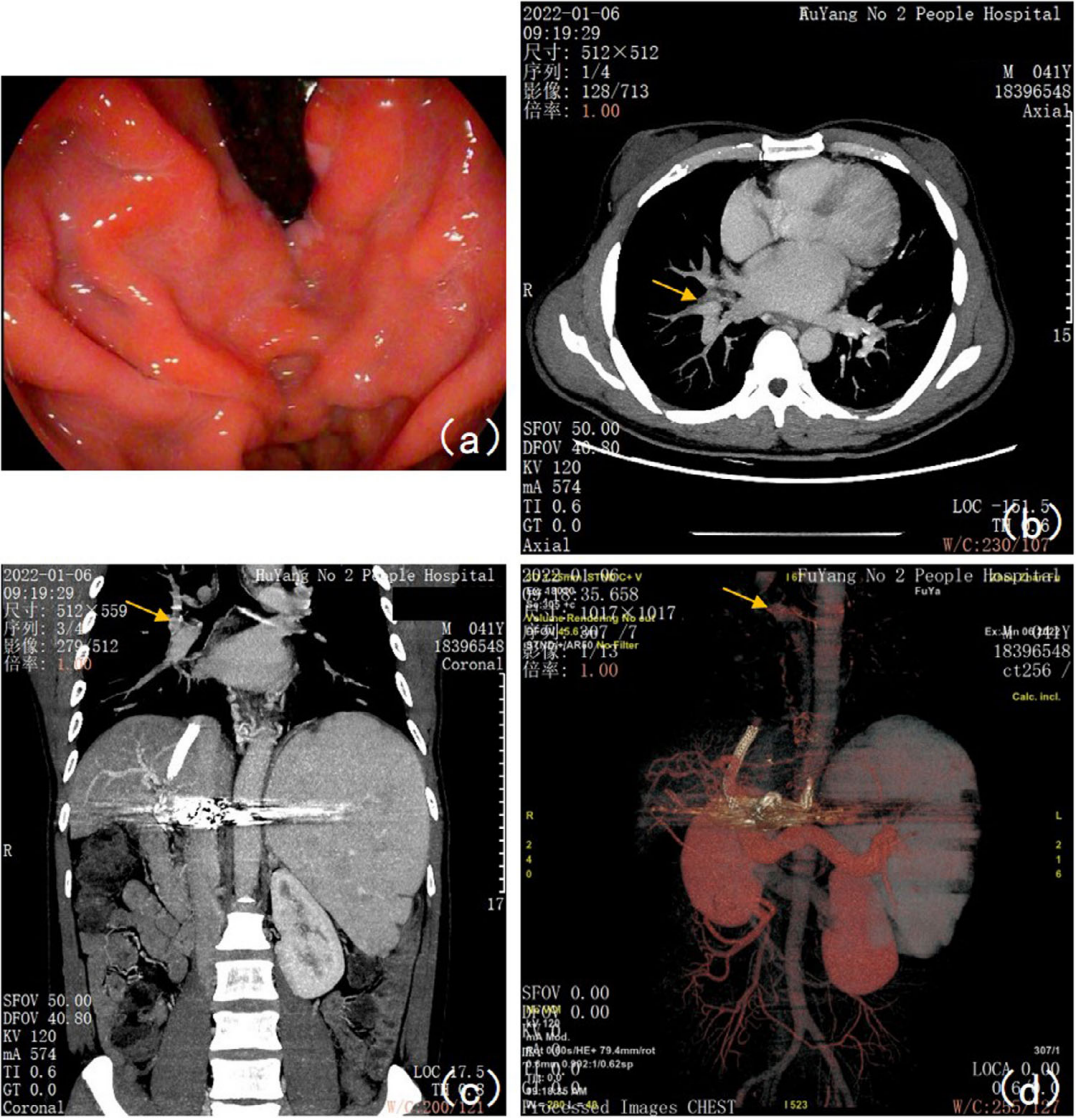
Gastroscopic review on January 26, 2021, and Portal vein imaging results on January 6, 2022: (a) Gastric fundal varices were significantly reduced with red sign (‐) and blood bubble sign (‐). (b) The cross‐sectional scan of the MIP image in the venous phase showed that the right hilar ectopic varices were significantly thinner than before (pointed by the yellow arrow in the figure). Enlarged breast glands on both sides (considered due to oral spironolactone). (c) The coronal scan of the MIP image in the venous phase showed that the right hilar ectopic varices were significantly thinner than before (pointed by the yellow arrow in the figure). (d) Portal venous reconstruction (VR) indicated thickened and tortuous esophageal and splenic veins, of which the wider lumen near the esophageal hiatus was 0.33 cm thick, and the main splenic vein was 1.93 cm thick, and tortuous vessels on both sides of the pulmonary hilar (pointed by the yellow arrow in the figure) that extended downward to connect to tortuous vessels around the esophagus and partially converged with the main portal vein, with a lumen thickness of 0.35 cm near the confluence section

A 40‐year‐old male with a 10‐year history of hepatitis B‐derived decompensated hepatic cirrhosis was admitted to our hospital on December 26, 2019, due to spitting 1000 ml of blood, and he manifested repeated hematemesis and melena during the hospitalization. He then received emergent endoscopic hemostasis treatments three times, that is, 1.5 ml tissue adhesive for gastric fundal varices + 14‐point esophageal variceal ligation, 1.5 ml tissue adhesive for gastric fundal varices, and 0.5 ml tissue adhesive for gastric fundal varices + 10 ml sclerosing agent for esophageal variceal sclerotherapy on December 26, 2019, January 6, 2020, and January 17, 2020, respectively. During hospitalization, the patient developed a fever. On January 16, 2020, chest CT showed an enlarged right hilar consolidation, and the patient tested positive for mycoplasma, suggesting mycoplasma pneumonia; therefore, azithromycin was prescribed as anti‐infective therapy, which relieved the fever. After discharge, the patient was generally in good condition, without further bleeding, and further received sequential endoscopic treatments for esophageal and gastric varices on April 8 (Figure 1a), April 15 (Figure 1b), August 19 (Figure 1c), and November 18 (Figure 1d), 2020. The enhanced chest CT scan performed on August 15, 2020, indicated an enlarged right hilum and showed a soft tissue mass near the right hilum, suggesting lymphadenopathy with the possibility of cancer. In addition, the patient had shortness of breath. Respiratory consultation led to a recommendation of bronchoscopy. However, because the patient showed significant hypersplenism and a persistent low platelet count (16 × 10^9^/L), bronchoscopy would carry a high risk of bleeding. Thus, on September 2, 2020, the patient underwent partial splenic artery embolization surgery under digital subtraction angiography. After surgery, the patient developed a fever, which was relieved with anti‐infection treatment; he was then discharged from the hospital on September 19. After discharge, the patient still felt short of breath and was readmitted on November 2, 2020. A chest CT scan performed on November 3 showed an enlarged right hilum, which was more enlarged than it had been on the CT scan performed on August 15. After admission, the patient received platelet‐increasing, acid‐suppressing, diuretic, and other symptomatic treatments. Bronchoscopy performed on November 11 indicated bronchial lumen stenosis in the right lower lobe and hypertrophic mucosa that also showed extrinsic compression changes and felt soft when touched with the cytology brush, and the bronchofiberscopic washing cytology and bronchoalveolar lavage fluid analysis results were unremarkable. The portal vein imaging (Figure 2a–c) performed on November 14 showed filling defects of the main portal vein, with a high possibility of thrombosis; portal hypertension with collateral circulation, esophageal varices, and partial projection of the bilateral hilar. Taking into account the patient's medical history, symptoms, and postadmission examination results, we considered that the patient's pulmonary hilar enlargement was ectopic varices caused by portal hypertension and recommended a transjugular intrahepatic portosystemic shunt (TIPS). The patient manifested worsened shortness of breath and was readmitted to our department on December 6, 2020, for TIPS therapy, which, together with gastric coronary vein embolization, was performed on December 20, 2020 (Figures 2d and 3a, Video S1,2). After surgery, the shortness of breath was significantly relieved. On January 6, 2021, a portography review indicated that pulmonary hilar ectopic varices were thinner diameters than before. (Figure 3b,c). On January 26, 2021, a gastroscopic review indicated that the esophageal and gastric fundal varices were thinner diameters than before and the red sign was negative. (Figures 3d and 4a). The patient had recurrent episodes of hepatic encephalopathy after TIPS, requiring hospitalization for each episode. On January 6, 2022, the re‐examination of portal vein imaging showed that the pulmonary hilar ectopic varices were further thinned than before (Figure 4b–d). The patient showed no symptoms of chest tightness and shortness of breath since a follow‐up.

## DISCUSSION

Essentially, ectopic varices occur in cases of portal hypertension or portal vein thrombosis when the portal circulation enters the systemic circulation through the collateral circulation branches between portal veins and the systemic circulation. The pathogenesis of ectopic varices mainly includes portal hypertension and abdominal adhesions.^4^ The causes of portal hypertension‐derived ectopic varices are mainly as follows: (1) intrahepatic portal hypertension, for example, hepatic cirrhosis, tumor invasion combined with the blockage of portal vein branches, hepatic vein thrombosis (with hypersplenism in some cases), and so forth; (2) extrahepatic portal hypertension, including portal veins (thrombosis or cancer embolus formation, spongelike changes, extrinsic compression) and splenic veins (thrombosis, extrinsic compression).^5^ These types of portal hypertension often cause duodenal varices, most commonly in the bulbous part, followed by the descending part. The mechanism of abdominal adhesion is that the formation of adhesions between the intestinal tract and the abdominal wall or other structures after abdominal surgery leads to the formation of collateral branches between the portal and systemic circulation. The patient in this report had varices in the bilateral pulmonary hilar caused by portal hypertension; this is the first report of such a case.

Vascular sources of esophageal‐gastric varices caused by portal hypertension are usually gastric coronary veins, short gastric veins, posterior gastric veins, and splenic veins. The portography of this patient showed that the esophageal and gastric varices originated from the gastric coronary vein shunt lumens, while the esophageal varices wound toward both hilar, giving rise to ectopic varices in the pulmonary hilar. We speculated that the possible causes of the patient's ectopic varices in the pulmonary hilar were as follows: (1) the formation of branch circulation on the side of portal hypertension caused by hepatitis B‐derived decompensated hepatic cirrhosis, (2) the aggravation of portal hypertension due to the formation of portal thrombosis, and (3) blockage of the esophageal and gastric fundal varices after repeated endoscopic treatment of esophagogastric varices, reduce the shunt lumens of the esophageal cavity, and intragastric varices, which caused further increases in the pressure on the portal veins and in turn increased the pressure on the extraesophageal varices, leading to the redistribution of collateral branches and, ultimately, to varices in the pulmonary hilar.

For ectopic varices treatment, there are currently neither guidelines nor unified treatment options recommended by expert consensus. Clinically, the goal is to prevent and treat the ectopic varices caused by gastrointestinal bleeding. Depending on the site, ectopic varices can have various clinical manifestations, such as repeated gastrointestinal bleeding, bladder bleeding, vaginal bleeding, and hematoperitoneum.^6^ In recent years, with improvements in detection techniques and tools, the incidence of ectopic varices has been increasing, and cases of ectopic varices caused death are also increasingly being reported; however, clinical cases of ectopic varices caused bleeding have only been reported sporadically and have been treated mainly with medication, endoscopic hemostasis, TIPS, and surgical intervention.^7^ Balloon‐occluded retrograde transvenous obliteration is considered a viable solution in managing ectopic varices hemorrhage especially when traditional techniques are unsuccessful or contraindicated.^8^ The disease is still understood through trial and error, making it difficult to clinically diagnose and treat. In the case reported here, due to bleeding in the digestive tract, the patient was repeatedly subjected to various treatments, including endoscopic tissue adhesive for esophageal and gastric varices, sclerotherapy, and esophageal variceal ligation within just over a year. After the endoscopic therapy, the patient gradually developed shortness of breath that could not be alleviated with medication. After the multidisciplinary team discussion among experts from various departments, including the respiratory, thoracic surgery, CT, and interventional therapy department, the patient was finally diagnosed with chest tightness and shortness of breath caused by tracheal compression due to varices in the pulmonary hilar derived from the hepatic cirrhosis‐related portal hypertension; consequently, he received the TIPS + gastric coronary vein embolization. After surgery, the shortness of breath was considerably alleviated, and the portography review indicated that the varices in the pulmonary hilar were significantly improved, with excellent treatment efficacy.

## CONFLICT OF INTEREST

The authors declare no conflict of interest.

## ETHICS STATEMENT

All procedures followed have been performed in accordance with the ethical standards laid down Declaration of Helsinki and its later amendments. And the written consent of the patient has been obtained.

## FUNDING INFORMATION

None.

## Supporting information


**Video S1**: Before the intrahepatic portosystemic shunt via the jugular vein, the pulmonary hilar varicose can be seen under X‐ray fluoroscopy with a contrast agent.

**Video S2**: After the intrahepatic portosystemic shunt via the jugular vein, the blood flow of the pulmonary hilar varicose shunt can be seen reduced under X‐ray fluoroscopy.
